# Supplementary material for: Stacking Model‐Based Classifiers for Dealing With Multiple Sets of Noisy Labels
Source: Biom J. 2025 Mar 12;67(2):e70042. doi: 10.1002/bimj.70042 (PMC11898607; doi:10.1002/bimj.70042)
Supplement: Supplementary file 1 — Supporting Information [file BIMJ-67-e70042-s001.zip › density-based-ensemble-model-main/competitors_real_data/netlab-master/netlab-master/help/demgmm3.htm]

Netlab Reference Manual demgmm3


# demgmm3

## Purpose

Demonstrate density modelling with a Gaussian mixture model.

## Synopsis

```
demgmm3
```

## Description

The problem consists of modelling data generated
by a mixture of three Gaussians in 2 dimensions with a mixture model
using diagonal covariance matrices. The priors are 0.3, 0.5 and 0.2; the
centres are (2, 3.5), (0, 0) and (0,2); the covariances are all axis aligned
(0.16, 0.64), (0.25, 1) and the identity
matrix. The first figure contains a scatter plot of the data.

A Gaussian mixture model with three components is trained using EM. The
parameter vector is printed before training and after training. The user
should press any key to continue at these points. The parameter vector
consists of priors (the column), and centres (given as (x, y) pairs as
the next two columns). The diagonal entries of the
covariance matrices are printed separately.

The second figure is a 3 dimensional view of the density function,
while the third shows the axes of the 1-standard deviation circles
for the three components of the mixture model.

## See Also

`gmm`, `gmminit`, `gmmem`, `gmmprob`, `gmmunpak`

---

**Pages:**
Index


---

Copyright (c) Ian T Nabney (1996-9)
